# Optimizing Expression of a Llama-Based Anti-PSMA Nanobody in *Escherichia Coli* and Its Application in Immunohistochemistry of Prostate Cancer Tissues

**DOI:** 10.34172/apb.025.45447

**Published:** 2025-12-23

**Authors:** Seyedeh Sheila Seyed-Motahari, Shahriyar Abdoli, Mohammad Ali Shokrgozar, Shiva Irani, Zahra Sharifzadeh

**Affiliations:** ^1^Department of Biology, SR.C., Islamic Azad University, Tehran, Iran; ^2^School of Advanced Medical Technologies, Golestan University of Medical Sciences, Gorgan, Iran; ^3^National Cell Bank of Iran, Pasteur Institute of Iran, Tehran, Iran; ^4^Department of Immunology, Pasteur Institute of Iran, Tehran, Iran

**Keywords:** *Escherichia coli*, Expression optimization, Immunohistochemistry, Nanobody, PSMA

## Abstract

**Purpose:**

Nanobodies possess unique properties that make them promising as tumor targeting agents. Prostate-specific membrane antigen (PSMA), overexpressed in prostate cancer, can be an excellent target for prostate cancer diagnosis. This study aimed to express and purify an anti-PSMA nanobody (PSMA-Nb) and assess its potential for detecting PSMA antigen in prostate cancer through immunohistochemistry (IHC).

**Methods:**

The PSMA-Nb gene was subcloned into pET-28a and expressed in *E. coli* Rosetta (DE3) and Rosetta-gami2 under varying IPTG/temperature/time conditions (0.5/1.0/1.5 mM; 16/30/37 °C; 4/16 h). The soluble and inclusion-body fractions were analyzed, and PSMA-Nb was purified via native Ni-NTA, confirmed by SDS-PAGE and anti-c-Myc Western blot. Binding was validated by ELISA against recombinant PSMA and by flow cytometry on LNCaP (PSMA+) and DU145 (PSMA-). For tissue studies, FFPE IHC quantified staining as fractional fluorescent area per mm^2^.

**Results:**

PSMA-Nb (~27 kDa) was enriched in *E. coli* inclusion bodies. A factorial screen identified Rosetta (DE3) with 1 mM IPTG at 37 °C for 16 h as the highest-expressing condition; resulting in a yield of approximately 84 mg/L. ELISA showed dose-dependent binding to recombinant PSMA, and flow cytometry confirmed antigen selectivity (LNCaP 68.5% vs. DU145 2.35%). IHC showed higher PSMA levels in tumor vs. normal tissue with both reagents (PSMA-Nb: 21.61±2.89 vs. 5.82±1.80; commercial antibody: 20.55±3.80 vs. 5.50±2.14; *P*<0.0001), with no difference between reagents (*P*=0.9624), supporting analytical validity.

**Conclusion:**

The superior features of nanobodies support antibody-based diagnostics in solid tumors. Purified PSMA-Nb detected PSMA on cancer cells and FFPE tissues, indicating a promising tool for prostate cancer diagnosis.

## Introduction

 Prostate cancer is regarded as the second most common type of cancer and the fifth major reason for cancer death in men, with around 1.4 million novel cases as well as 375,000 deaths in the world. The five-year relative survival rates for metastatic and localized prostate cancers are 30% and 100%, respectively. Global variations in the incidence rates are largely attributable to differences in the use of various diagnostic testing methods.^1^ The detection time and the staging of primary, metastatic, and relapsed prostate cancers are extremely important for the management of prostate cancer.^2^ Early diagnosis, and management of prostate cancer, have recently undergone significant advances, with increasing evidence highlighting both strengths and weaknesses of different detection assays.

 PSMA is a type II membrane glycoprotein with 750 amino acids, and a molecular weight of about 100 kDa after glycosylation.^3^ Poorly differentiated and metastatic prostate cancers show high levels of PSMA expression; making it a valuable marker for prostate tumor cells. PSMA expression is mostly prostate-specific, with very low levels identified in the duodenum, kidney, salivary glands, neuroendocrine system, and proximal renal tubules. As PSMA expression is* higher *in most prostate cancer cells when compared to normal tissues, it was chosen as a target for Prostascint, a Food and Drug Administration (FDA)-approved monoclonal antibody (mAb) for imaging prostate cancer.^4^ Furthermore, several monoclonal antibodies have been developed that can bind to a particular epitope in the extracellular domain of PSMA, offering considerable potential for the diagnosis and treatment of prostate cancer.^3,5^

 Conventional antibodies can selectively detect tumor cell antigens, but their pharmacological efficacy is typically constrained by their large size, high cost and labor-intensive manufacturing, and immunogenicity. Variable heavy chain of heavy-chain-only antibodies (VHHs), also known as nanobodies, naturally obtained from camels and llamas, are regarded as the smallest antibody fragments which can maintain the binding affinity to their targets.^6^ Their distinctive paratope architecture and monomeric single-domain nature enable recognition of haptens and cryptic epitopes that are inaccessible to classical antibodies. Moreover, nanobodies are beneficial for *in vitro* and *in vivo* applications because of their low immunogenicity, high stability, enhanced solubility, and low production cost.^7^ Their single-domain nature, also facilitates straightforward conjugation to diverse proteins, reporter molecules, or therapeutic agents.^8^

 Nanobodies have been expressed in different microorganisms such as *Escherichia coli* (*E. coli*), Lactobacillus, *Pichia pastoris, *and *Saccharomyces cerevisiae *due to their small size and lack of glycosylation. Additional expression systems such as insect cell lines, yeasts, mammalian cells, and transgenic plants have also been used for efficient expression of nanobodies.^7,9,10^ However, among these methods, prokaryotic expression systems remain particularly advantageous, as they allow nanobodies to be easily produced in effective and functional recombinant. Because of its well- characterized genetics, inexpensive cultivation, high growth rate, and ease of manipulation, *E. coli *is the preferred host organism for the production of recombinant proteins.

 A previously developed PSMA-specific nanobody (PSMA-Nb) has demonstrated high PSMA-targeting capability and favorable pharmacokinetics in SPECT-CT studies.^11^ Given that PET scanning requires expensive equipment and highly trained personnel, and that immunohistochemistry (IHC), a widely accessible method in most pathology laboratories, has not yet been adapted for use with PSMA-Nb, it would be valuable to evaluate the potential of PSMA-Nb for detecting PSMA antigen in prostate cancer tissue via immunohistochemistry.

 In this study, we investigated the ability of *E. coli *as the host organism to express the PSMA-Nb and optimized the various culture conditions for enhancing the recombinant protein expression. Then, the binding potential of the purified nanobody to PSMA-expressing cells was evaluated by ELISA and flow cytometry. Finally, as a proof of concept, its ability to detect PSMA expression in human PCa was evaluated by IHC.

## Methods

###  Strains, plasmids, and cell lines

 E. coli expression pET-28a (Novagen, USA) plasmid was used for cloning the PSMA-Nb. E. coli Top10, Rosetta (DE3), and Rosetta Gami2 strains (Pasteur Institute of Iran, Tehran, Iran) were used for recombinant protein expression. The PSMA-positive LNCaP (androgen-sensitive human prostate adenocarcinoma) and PSMA-negative DU145 (human prostate carcinoma) cell lines (Pasteur Institute of Iran, Tehran, Iran) were used for the verification of recombinant nanobody binding. Both cell lines were cultured in DMEM high glucose medium supplemented with 10% fetal bovine serum, 2 mM Glutamine, 100 U/ml penicillin, and 0.1 mg/ml streptomycin at 37 °C in a humidified incubator with 5% CO₂. The cell culture medium and the supplements were obtained from the Biosera Company, France.

###  Reagents 

 The restriction endonucleases *EcoRV*, *BamHI and XhoI, *T4 ligase, and isopropyl-β-D-thiogalactopyranoside (IPTG) were purchased from ThermoFisher Scientific (Ottawa, Canada). The 10 kb DNA marker was purchased from Sangon Biotech Co., Ltd. (Shanghai, China) and the protein marker was from Thermo Fisher Scientific (Ottawa, Canada). Anti-c-myc mAb conjugated to horseradish peroxidase (HRP) was obtained from Roche (Mannheim, Germany). Nickel-NTA agarose resin was purchased from ABT (Madrid, Spain). DMSO, lysozyme, and phenylmethylsulfonyl fluoride (PMSF) were obtained from Biobasic (Toronto, Canada). All used reagents had an analytical grade and were obtained from Sigma-Aldrich (St Louis, MO, USA).

###  Synthesis of the recombinant PSMA-Nb and cloning into pET-28a 

 The PSMA-Nb (JVZ-007) was kindly provided by Dr. W.M. van Weerden ^11^. The c*- myc and polyhistidine tags* were fused to the *C*-*terminus* of PSMA-Nb for ease of *detection* and *purification, respectively*. The final PSMA-Nb sequence was codon-optimized, synthesized, and cloned into a pUC vector. Then, the PSMA-Nb was digested with *BamHI and XhoI*restriction endonucleases, gel extracted, and subcloned into similarly digested ends of the pET-28a vector. The ligation mixture was transformed into *E. coli *Top10 cells, and the final construct was sequenced.

###  Expression of recombinant PSMA-Nb

 The PSMA-Nb was expressed in two different *E. coli* strains, Rosetta (DE3) and Rosetta Gami2. Briefly, about 100 ng of pET-28a-PSMA-Nb was added to Rosetta (DE3) and Rosetta Gami2 competent cells. Then, a single colony was inoculated into 3 ml of LB broth, which contained 50 μg/ml kanamycin. The culture was then shaken at 37 °C overnight and transferred to Kanamycin-containing LB medium at a 1∶10 ratio. Once the cell’s cultures reached optimal density at 600 nm (OD_600_) of 0.6-0.8, the PSMA-Nb expression was induced by adding 1 mM IPTG, followed by shaking for another 16 h under the same conditions. The cell pellets were harvested by centrifugation at 10,000 × g for 5 min. To optimize the PSMA-Nb expression, cultivations were performed under various conditions, such as different IPTG concentrations (0.5/1.0/1.5 mM), temperatures (16/30/37 °C), and induction times (4/16 h). (Workflow schematic is provided in Supplementary Table S1).

###  SDS-PAGE analysis

 Following centrifugation, the total bacterial pellet was lysed (lysis buffer = 100 mM NaH_2_PO_4_, 10 mM urea, pH = 8). Protein samples were prepared in a gel loading buffer (0.25 M Tris-HCl, pH 6.8, 5% glycerol, 5% 2-mercaptoethanol, 3% sodium dodecyl sulfate (SDS), and 0.2 mg/mL bromophenol blue) as the sample buffer to solubilize the protein samples. Non-induced and induced cell samples were then heated to 95 °C for 5 min to denature proteins. The samples were centrifuged at 15,000 × g for 1 min, loaded equally (1 µg per sample) on a 12% SDS-PAGE gel, and run at 150 V in Tris-Acetate/EDTA (TAE) buffer.

###  Determination of PSMA-Nb protein solubility

 For the solubility assessment of recombinant PSMA-Nb, the cell pellet of bacterial cultures was harvested by centrifugation for 15 min at 10,000 × g and resuspended in a lysis buffer (50 mM NaH_2_PO_4_, 300 mM NaCl, and 10 mM imidazole). Cell lysis was performed by incubating the culture with 1 mg/mL lysozyme for 30 min on ice, followed by sonication (30% amplitude, 6 × 10 s with 20 s pauses at 200-300 W) on ice. The sonicated samples were then centrifuged at 10,000 × g for 25 min at 4 °C. The presence of the PSMA-Nb protein was examined in the supernatant (soluble fraction) and pellet fractions (inclusion bodies) by SDS-PAGE analysis. All soluble (S) and insoluble/inclusion-body (IB) fractions were derived from the same culture and the same lysate processed in parallel. Following cell lysis, a single centrifugation step separated the supernatant (S) from the pellet (IB), with upstream growth/induction/lysis conditions and downstream handling/volumes were identical for both fractions. The supernatant and the pellet were analyzed as the soluble and insoluble fractions, respectively.

###  Western blot analysis

 The nanobody expression was subjected to the western blotting. Following centrifugation, the total bacterial pellet was prepared as explained above; then bacterial lysate was separated by 12% SDS-PAGE and transferred to polyvinylidene fluoride (PVDF) membrane electrophoretically. The membrane with transferred proteins was blocked overnight using 3% bovine serum albumin (BSA) in Tris-buffered saline (TBS) with 0.1% Tween 20 and cut into two pieces. One membrane was incubated for 1 h with a 1:1000 dilution of anti-c-myc-HRP mouse monoclonal antibody conjugated to horseradish peroxidase. Another piece of the membrane was subjected to incubation with a 1:1000 dilution of HRP-labeled anti-His antibody. After washing, 3,3’-diaminobenzidine (DAB) solution was added and incubated at room temperature until color development occurred (about 10 min in the dark).

###  Purification of recombinant PSMA-Nb

 Rosetta (DE3) bacteria (harboring pET-28a-PSMA-Nb) was induced with 1 mM IPTG and incubated for 16 h at 37 °C. The cell pellets were collected at 4000 × g for 15 min and then suspended in 3 ml of PBS with 1 mM PMSF. Afterward, they were lysed by sonication and the mixture was centrifuged at 15000 × g for 25 min at 4 °C. The supernatant was loaded on a nickel-NTA agarose column, followed by serial washing with buffers that contained 10- and 20-mM imidazole, respectively. Finally, the nanobody was eluted with the same buffer, with increasing imidazole concentrations of 100, 300, and 500 mM. The purified nanobody was dialyzed against 10 mM PBS (pH 7.2) for 24 h, and its concentration was measured through the Bradford assay. (Workflow schematic is provided in Supplementary Table S1).

###  Confirmation of PSMA binding by ELISA

 A 96-well microplate was coated with 1 μg/well of PSMA antigen in 100 μL of coating buffer (0.1 M Na_2_CO_3_, 0.1 M NaHCO_3_, pH 9.5) and incubated overnight at 4°C. Wells coated with BSA antigen served as negative controls. Then, the wells were rinsed three times with PBST buffer (PBS with 0.05% tween-20), followed by blocking with 5% skimmed milk in PBS (MPBS), and incubation for 1 h at 37 °C. After washing three times with PBST buffer, different concentrations of PSMA-Nb (1, 3 and 5 μg/ml) were added to separate wells and incubated for 1 h at room temperature. Wells were then incubated with, 100 μl of anti-c-myc mouse antibody conjugated with HRP was added to each well (1:1000 dilution). After 1 h of incubation at room temperature, 100 μL of tetra-methyl benzidine (TMB) was added as the substrate. Optical density was measured at 450 nm after stopping the reaction with 3N sulfuric acid.

###  Flow cytometry

 The binding activity of PSMA-Nb to prostate cancer cells was assessed by flow cytometry technique. Both cell lines were incubated with FITC-Labeled monoRab^TM^ rabbit anti-camelid VHH antibody for 40 min at 4 °C. Flow cytometry was performed using a Partec PAS-III (Partec GmbH, Munster, Germany) system. The FlowJo 7.6.1 software was used for data analysis.

###  Paraffin block preparation

 The study included samples from nine patients diagnosed with prostate cancer and nine healthy individuals as the control group. To confirm the cancer diagnosis of the biopsies obtained from the prostate, the samples were stained with Hematoxylin and Eosin (H&E) and examined by two experienced pathologists at Hashemi Nezhad Hospital. All tissues were formalin-fixed, paraffin-embedded (FFPE) to create FFPE blocks.

###  PSMA Immunohistochemistry

 FFPE tissue sections (5 μm thick) were mounted on Superfrost Plus slides (Fisher Scientific), dewaxed, and rehydrated through a series of graduated ethanol washes for subsequent immunostaining.

 A rabbit polyclonal anti-PSMA antibody (PSMA1, Biorbyt, 1:100) served as a positive control. All normal and tumor sections were heated in a microwave oven (800 W) for 20 minutes with TBS buffer. Then, sections were blocked with serum-free protein blocking (Agilent) for 30 min. PSMA-Nb (1 and 3 μg) and PSMA1 antibody (1:100) were applied for 24 h at 2-8 °C. The sections were rinsed three times with PBS. Then, monoRab^TM^ rabbit anti-camelid VHH Cocktail (FITC conjugated, Genscript) and goat anti-rabbit IgG (H + L) antibody (FITC conjugated, Biorbyt) mouse antibody and were subsequently added to PSMA-Nb- and PSMA1-incubated sections, respectively, and incubated for 1 h at room temperature in the dark place. Next, all the sections were counterstained with DAPI (Sigma-Aldrich), washed with PBS, and mounted with glycerol and PBS solution. Lastly, an Olympus microscope was used to photograph the results. The IHC results were quantified by dividing the total fluorescent area by the total area of each section to produce the average protein level per mm^2^. (Workflow schematic is provided in Supplementary file, Table S1).

###  Statistical Analysis

 For the ELISA dose–response experiments, group differences were assessed by one-way ANOVA with Tukey’s multiple comparisons test. For tissue expression analysis, two-sided unpaired t-tests were run to compare tumor versus normal for each antibody. Statistical significance was set at *P* < 0.05. All analyses and data visualization were performed using GraphPad Prism v10.4.1 (GraphPad Software, San Diego, CA, USA). Standardized effect sizes were computed as Hedges *g* using the two-sample pooled-SD formulation with the Hedges small-sample correction (*J*); 95% confidence intervals for *g* were obtained via nonparametric bootstrap or large-sample approximations.

## Results

###  Construction of pET-28a-PSMA-Nb

**Figure 1:**
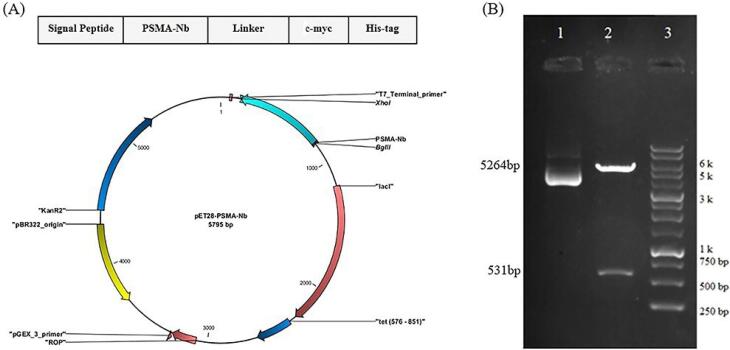
Design and construction of pET-28a-PSMA-Nb plasmid. (A) Schematic representation of pET-28a expression vector harboring the gene encoding PSMA-Nb. The construct contains c-myc and his-tag sequences for nanobody detection and purification, respectively. (B) Restriction enzyme analysis of the recombinant pET-28-PSMA-Nb expression vector. Lane 1: undigested vector (5795bp); Lane 2: vector digested with *BamHI and XhoI* restriction enzymes (5264 bp and 531 bp bands); Lane 3: 1 Kb DNA. DM3100 ExcelBand^TM^ 1 KB (0.25–10 kb) DNA Ladder is composed of 13 individual DNA fragments: 250, 500, 750, 1k, 1.5k, 2k, 2.5k, 3k, 4k, 5k, 6k, 8k, 10k bp

**Figure 2:**
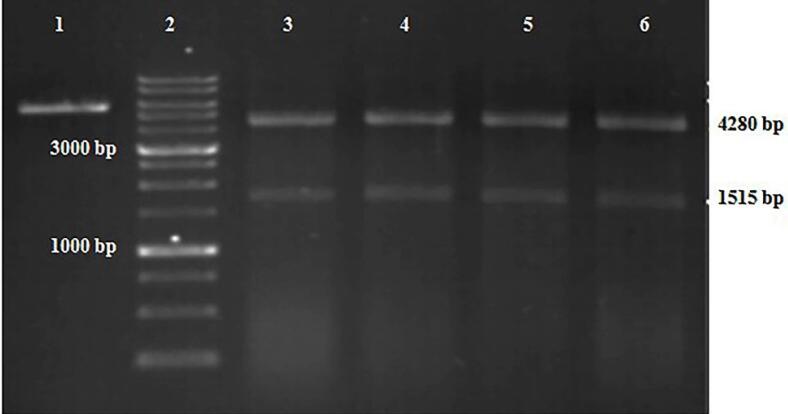
Confirmation of Top10 competent cells transformation. Lane 1: pET-28a without insert (negative control); Lane 2: 1 Kb DNA marker; Lanes 3-6: recombinant plasmids from four bacterial colonies digested with *EcoRV*. DM3100 ExcelBand^TM^ 1 KB (0.25–10 kb) DNA Ladder is composed of 13 individual DNA fragments: 250, 500, 750, 1k, 1.5k, 2k, 2.5k, 3k, 4k, 5k, 6k, 8k, 10k bp

 As seen in Figure 1A, the PSMA-Nb gene was subcloned in the expression vector pET-28a, and the recombinant plasmid was confirmed by restriction enzyme analysis. The results of gel electrophoresis displayed bands of 5264bp and 531 bp when digestion of recombinant plasmid was done with *BamHI and XhoI* (Figure 1B). Moreover, nucleotide sequencing proved the correct sequence of the PSMA-Nb gene. The final construct was transformed into Top10 competent cells and confirmed by *EcoRV* restriction enzyme digestion (Figure 2).

###  Optimization of PSMA-Nb expression 

**Figure 3:**
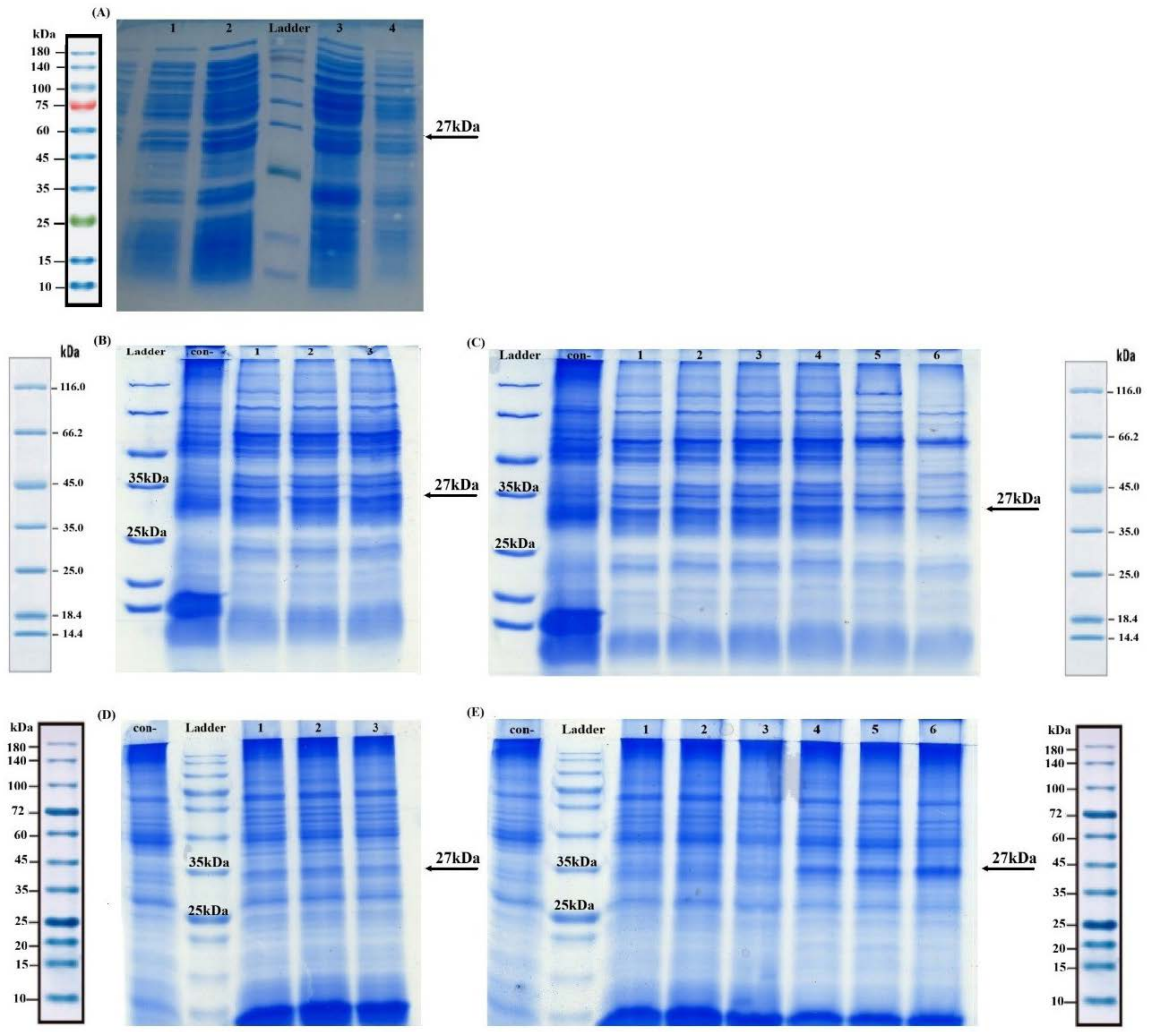
SDS-PAGE analysis of PSMA-Nb expression at different culture conditions. A. Total protein was extracted from *Escherichia coli* Rosetta (DE3) and Rosetta Gami2 (left side and right side of the ladder, respectively) containing pET-28a-PSMA-Nb plasmid before (lanes 1 and 4) and after (lanes 2 and 3) induction with 1 mM IPTG for 16 h at 37˚C. Lane M (ladder): SMOBIO ExcelBand^TM^ 3-color Regular Range, PM2510, 10–180 kDa; marker bands at 10, 15, 20, 25 (green), 35, 45, 60, 75 (red), 100, 140, 180 kDa. B-E. Optimization of PSMA-Nb expression after 4 h (B, C) and 16 h (D, E). B, D. Con-: negative control; Lanes 1-3: protein expression at 16 ºC after induction with 0.5, 1, and 1.5 mM IPTG, respectively. C, E. Con-: negative control; Lanes 1-3: protein expression after induction at 30 ºC with 0.5, 1, and 1.5 mM IPTG, respectively. Lanes 4-6: protein expression after induction at 37 ºC with 0.5, 1, and 1.5 mM IPTG, respectively. The band between 25 kDa and 30 kDa corresponds to an PSMA-Nb (27kDa). Lanes B and C: Bio Basic^TM^ unstained protein molecular weight marker (14.4–116.0 kDa), with discrete bands at ~14.4, ~18.4, ~25, ~35, ~45, ~66.2, and ~116 kDa. This marker is optimized for use with 12% Tris-Glycine SDS-PAGE gels. Lanes D and E: SMOBIO ExcelBand^TM^ all blue regular range plus protein marker (PM1600, 10–180 kDa), containing blue-stained bands at ~10, ~15, ~20, ~25, ~35, ~45, ~60, ~72, ~100, ~140, and ~180 kDa

 The pET-28a-PSMA-Nb plasmid was transformed into two *E. coli* strains including Rosetta (DE3), and Rosetta Gami2 competent cells. Analysis of protein expression revealed bands at the expected molecular weight (27 kDa) for both strains (Figure 3A). Quantification using ImageJ revealed that PSMA-Nb expression in Rosetta Gami2 was considerably lower than Rosetta (DE3), with fold-changes of 3.94 and 5.78, respectively. Subsequently, PSMA-Nb expression at various IPTG concentrations (0.5, 1, and 1.5 mM) and incubation temperatures (16, 30, and 37 ° C) was evaluated in Rosetta (DE3). Increasing the expression temperature from 16 °C to 37 °C enhanced the recombinant protein expression across all tested IPTG concentrations. At 1 mM IPTG, fold-changes in protein concentration were 4.6, 5.96, and 7.13 at 16 °C, 30 °C, and 37 °C, respectively (Figure 3). The maximum PSMA-Nb expression was observed with Rosetta (DE3) cultures induced with 1 mM of IPTG at 37 ºC after 16 h.

###  Evaluation of protein solubility

**Figure 4:**
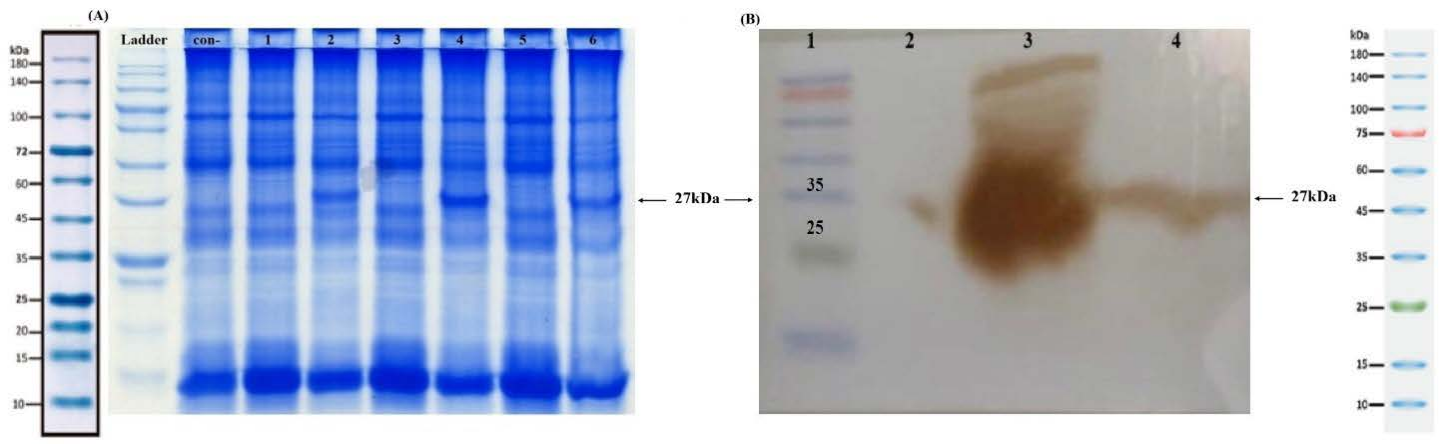
Protein solubility assessment. A. SDS-PAGE analysis of PSMA-Nb expression in soluble and insoluble fractions. Con-: Uninduced Rosetta (DE3) cells (negative control), Lanes 1, 3, and 5: soluble fractions of PSMA-Nb after induction with 0.5, 1, and 1.5 mM IPTG, respectively. Lanes 2, 4, and 6: Insoluble fractions of PSMA-Nb after induction with 0.5, 1, and 1.5 mM IPTG, respectively. The incubation of all samples was done at 37 °C for 16 hours. Ladder lane: SMOBIO ExcelBand^TM^ All Blue Regular Range Plus Protein Marker (PM1600, 10–180 kDa), containing blue-stained bands at ~10, ~15, ~20, ~25, ~35, ~45, ~60, ~72, ~100, ~140, and ~180 kDa. B. Western blot analysis. Lane 1: Protein marker, Lane 2: Uninduced Rosetta (DE3) cells (negative control), Lane 3: PSMA-Nb expression in inclusion bodies (insoluble fractions), Lane 4: PSMA-Nb expression in soluble fractions. The protein band around 27kDa corresponds to PSMA-Nb. Lane 1 (Ladder): SMOBIO ExcelBand^TM^ 3-color Regular Range (PM2500), 10–180 kDa; marker positions at 10, 15, 20, 25 (green), 35, 45, 60, 75 (red), 100, 140, 180 kDa (25 and 35 kDa are indicated on the image). The soluble (S) and insoluble/inclusion-body (IB) fractions were derived from the same culture/lysate and processed in parallel. After lysis, a single centrifugation step separated supernatant (S) from the pellet (IB); growth/induction/lysis conditions and handling/volumes were identical for both fractions.

 The protein solubility assessment showed that the highest PSMA-Nb concentration was achieved in insoluble inclusion bodies in Rosetta (DE3). The fold change in PSMA-Nb levels at 1 mM IPTG was 21.60 in the insoluble fraction and 2.51 in the soluble fraction (Figure 4A). Western blot analysis using anti-c-myc antibody confirmed that the PSMA-Nb was primarily expressed as inclusion bodies (Figure 4B).

###  Purification of PSMA-Nb protein and western blotting

**Figure 5:**
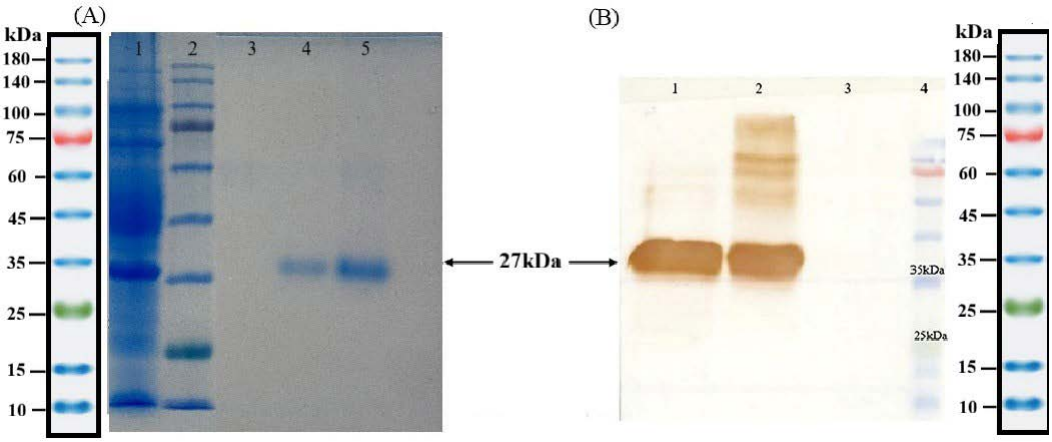
SDS-PAGE and western blot analysis of PSMA-Nb purified by Ni-NTA column. A. Lane 1: Flow through; Lane 2: Protein marker; Lane 3: control negative; Line 4: Elution 1 (100 mM imidazole); Lane 5: Elution 2 (300 mM imidazole). B. Western blot analysis of PSMA-Nb; Lane 1: Elution 3 (500 mM imidazole); Line 2: Elution 2 (300 mM imidazole); Line 3: control negative; Line 4: Protein marker. Ladder lane (for both panels): SMOBIO ExcelBand^TM^ 3-color regular range (PM2510), 10–180 kDa; marker bands at 10, 15, 20, 25 (green), 35, 45, 60, 75 (red), 100, 140, 180 kDa (25 and 35 kDa are indicated on the images)

 Nanobodies were purified by applying Ni-NTA chromatography following the Qiagen purification protocol; then the fractions were analyzed by SDS-PAGE and Western blotting. As shown in Figure 5A, the highest yield of protein was obtained in elution buffer 3, which contained 500 mM imidazole*. Western blot analysis with *anti-c-myc antibody confirmed the correct *expression* of the PSMA-Nb, revealing a protein band in the desired range with a molecular weight of about 27 kDa (Figure 5B).

###  Antigen binding activities of PSMA-Nb 

**Figure 6:**
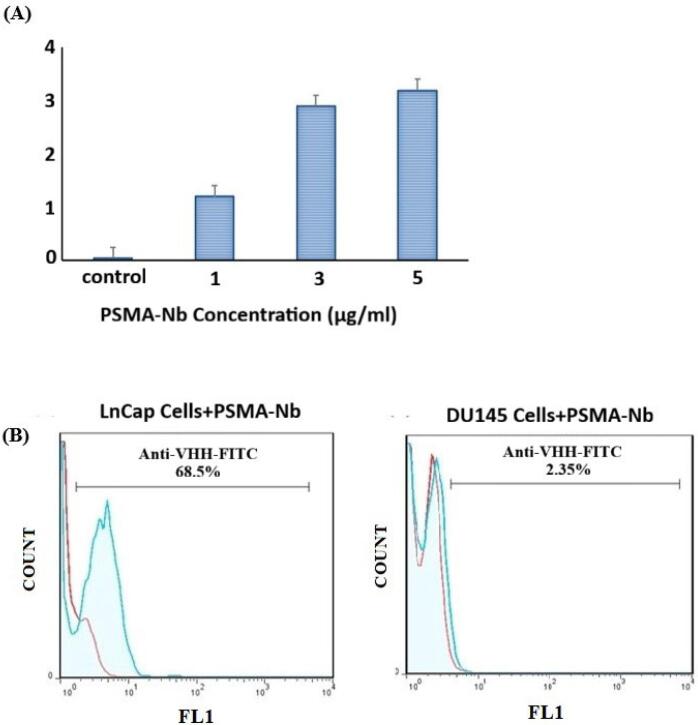
ELISA and flow cytometry analysis of the specific binding of PSMA-Nb to PSMA. A) ELISA-based PSMA detection with varying concentrations of PSMA-Nb. Specific binding to PSMA was observed, while BSA showed no binding. Optical density (OD) increased with nanobody concentration, reaching a plateau beyond 3 μg/mL. OD at 1, 3, and 5 μg/mL was significantly higher than control (one-way ANOVA with Tukey’s post-hoc test; p = 0.0004, < 0.0001, < 0.0001, respectively). OD at 3 and 5 μg/mL exceeded 1 μg/mL (p < 0.0001) but did not differ from each other (p = 0.1011). B) The purified PSMA-Nb protein could successfully recognize PSMA-positive LNCaP cells (left side); Du145 cells (right side) were used as the negative control. The PSMA-positive fraction was 68.5% in LNCaP versus 2.35% in DU145, confirming nanobody specificity

 To confirm the correct folding and functionality of the purified PSMA-Nb, we performed ELISA and flow-cytometry assays. ELISA showed a concentration-dependent increase in OD (Figure 6A), with values at 1, 3, and 5 μg/mL significantly above control (one-way ANOVA with Tukey’s test, adjusted *P* = 0.0004, < 0.0001, < 0.0001, respectively); 3 and 5 μg/mL exceeded 1 μg/mL (both *P* < 0.0001), whereas 3 vs. 5 μg/mL was not significant (*P* = 0.1011), indicating a plateau beyond 3 μg/mL. Flow cytometry confirmed specificity, revealing a PSMA-positive population of 68.5% (LNCaP) vs 2.35% (DU145) (Figure 6B).

###  Immunohistochemistry analysis of PSMA expression at prostatic biopsy

**Figure 7:**
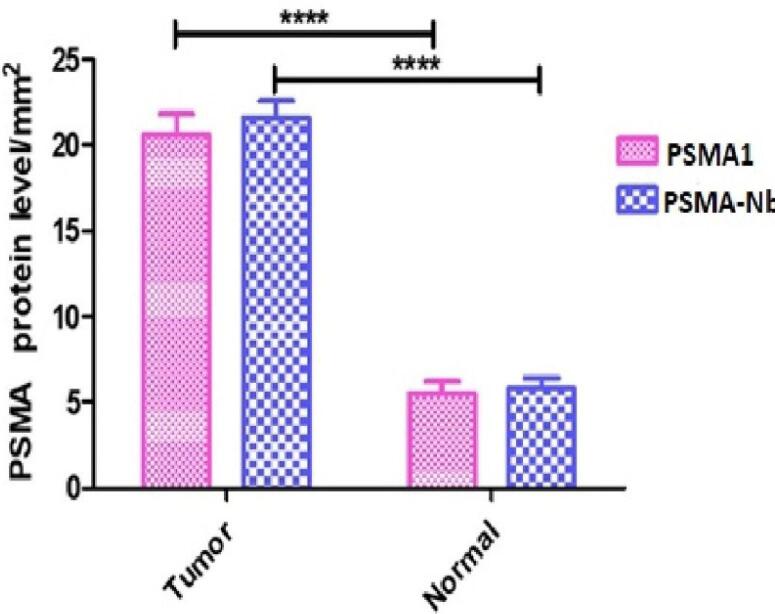
Prostate cancer and normal cell IHC staining. Fluorescence signals from prostate tissues stained with: A) 1 µg/ml of PSMA-Nb, B) 3 µg/ml of PSMA-Nb, and C) commercial anti-PSMA antibody (1 µg/ml of PSMA1). The PSMA antigen was stained with PSMA-Nb and anti-VHH FITC, respectively, (first column) and the cell nuclei in the tissues were stained blue with DAPI (second column). Overlay images between green fluorescent protein and DAPI-positive nuclei (third column) show prostate cells expressing PSMA antigen. In each image, the higher amount of green indicates higher protein expression, which is calculated quantitatively by the ImageJ software. (Objective 20 × ; scale bar 100 µm; identical exposure and processing across panels).

 The diagnostic potential of the purified PSMA-Nb was evaluated by IHC and the immunohistochemical images were analyzed by ImageJ software. The cell nuclei in the tissues were counterstained with DAPI (blue), and the expression level of the target protein (green) in the cells was detected by PSMA-Nb. In each image, the higher amount of green indicates the higher PSMA protein expression. As can be seen in Figure 7, PSMA expression was markedly higher in tumor tissues compared to normal ones. Since the lower concentration (1 µg/ml) was almost as good as the 3 μg/ml concentration, this quantity of PSMA-Nb was used for all samples of tumor and normal sections.

**Figure 8:**
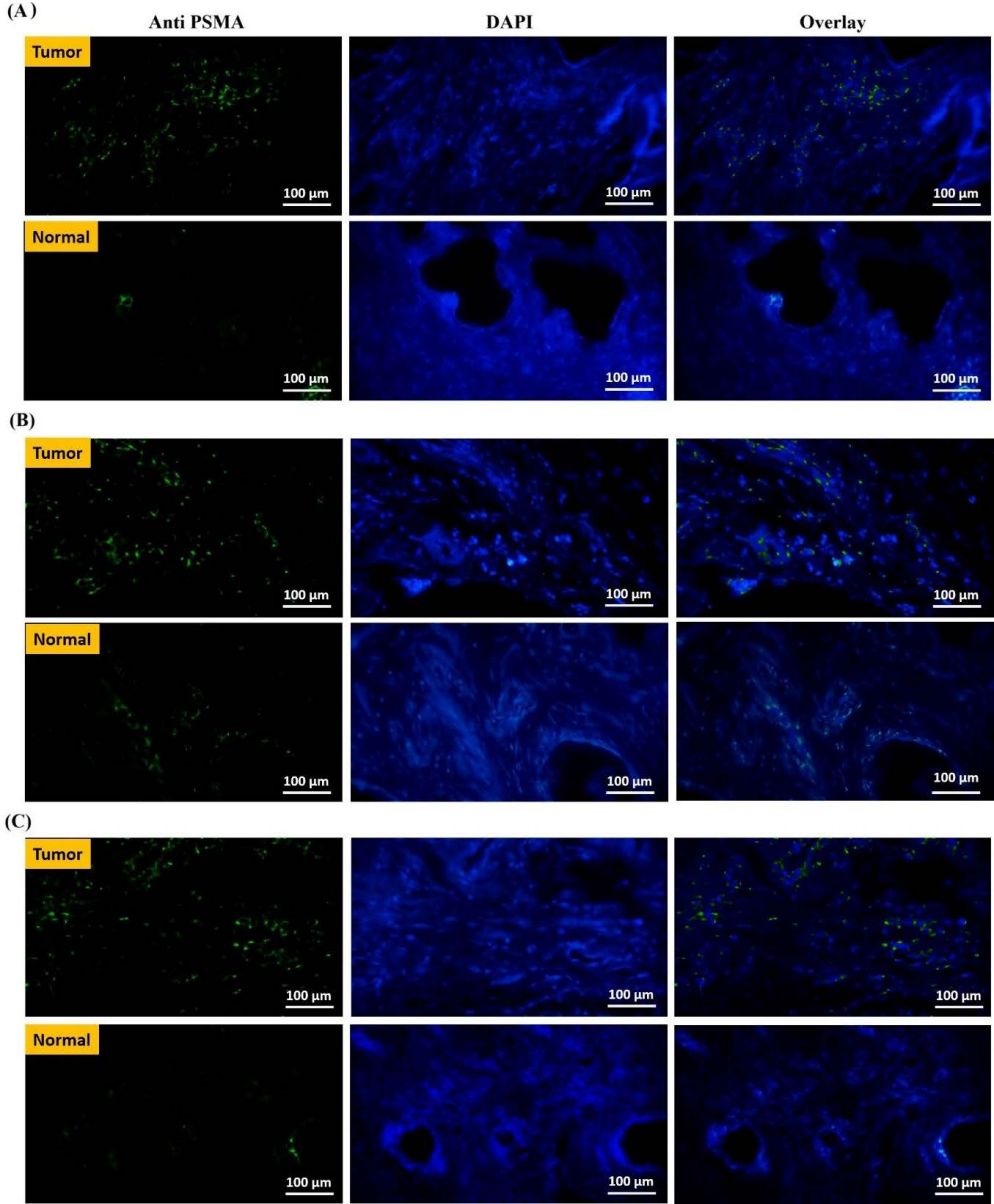
Data from the ImageJ immunohistochemical staining on nine cancerous and nine normal tissues. The chart shows a significant difference in PSMA antigen surface expression levels between normal and tumor tissues from prostate cancer biopsies (6 vs. 22 per mm^2^). Evaluation of PSMA antigen expression using the recombinant PSMA-Nb showed significantly higher levels in tumor tissue (21.61 ± 2.89) compared with normal tissue (5.82 ± 1.80), with strong statistical significance (*P* < 0.0001). Evaluation of PSMA antigen expression using the commercial PSMA1 antibody demonstrated significantly higher levels in tumor tissue (20.55 ± 3.80) compared with normal tissue (5.50 ± 2.14), with strong statistical significance (*P* < 0.0001). Direct comparative FFPE-IHC quantification shows equivalent PSMA detection by the recombinant PSMA-Nb and the commercial antibody, with no significant difference in measured expression (*P* = 0.9624)

 In matched tumor–normal sections, PSMA-Nb yielded 21.61 ± 2.89 versus 5.82 ± 1.80 (*P* < 0.0001; ≈3.7-fold difference), and the commercial antibody showed 20.55 ± 3.80 versus 5.50 ± 2.14 (*P* < 0.0001; ≈3.7-fold) (Figure 8). A paired analysis revealed no inter-reagent difference (*P* = 0.9624), indicating equivalent staining intensity and diagnostic performance. Using the same predefined IHC threshold, both reagents correctly identified all tumor and normal samples, corresponding to 100% sensitivity and 100% specificity within this proof-of-concept cohort. Based on the observed mean difference (~15 units) and pooled SD (~2.5), the standardized effect size (Hedges g) was ~6.25 for PSMA-Nb and ~4.65 for the commercial antibody, which fall in the ‘huge’ range per conventional benchmarks, indicating an exceptionally strong tumor–normal contrast and high statistical power even with the current sample size. These results confirm that the recombinant PSMA-Nb performs comparably to the commercial antibody for FFPE-IHC–based PSMA detection.

## Discussion

 More than three decades have passed since the FDA approved the first therapeutic antibodies, which were murine-derived mAbs. Today, there are more than 100 FDA-approved antibodies on the market. The monoclonal antibody industry is worth 145 billion dollars, which is growing at an 11% annual rate. These new drugs have been approved to treat diverse human diseases, including different cancers, autoimmune, metabolic, as well as infectious diseases.^12^

 Nanoscale VHHs, also known as nanobodies, are the smallest, naturally-derived antigen-binding fragments that have full antigen-binding potential with high affinity for their targets.^13,14^ Because nanobodies have longer complementarily determining region (CDR) domains than traditional antibodies, they are more sensitive to the detection of tumor-associated cell surface antigens.^15-17^ In addition to having a long shelf life at 4 °C and 20 °C, nanobodies can also resist high temperatures, elevated pressures, non-physiological pHs (3.0-9.0), and even chemical denaturants with the highest strength, all while preserving their ability to bind antigens.^18-20^ These characteristics position nanobodies as a highly attractive candidate for a wide range of biotechnological applications, especially for the specific, accurate, and efficient targeting of tumors *in vivo*.^10^ Additionally, nanobodies can be chemically conjugated to pharmaceuticals, nanoparticles, and radionuclides. They can also be genetically fused to Fc-domains, peptide tags, other nanobodies, or toxins. Nanobodies can be useful for tumor molecular imaging because of their enhanced tumor penetration, high tumor-to-background ratio, minimal off-target retention as well as rapid renal clearance. Since nanobodies can target inaccessible tumor markers and offer different delivery options, they are well suited for intracellular targeting, a property that is not possible with mAbs.^14^

 PSMA, a tumor-associated antigen with high expression in cancer cells, is amenable to being targeted by specific antibodies. Its expression level correlates with disease aggressiveness, underscoring its clinical importance in prostate cancer. PSMA-binding nanobodies can identify PSMA-expressing prostate tumors with considerable specificity.^21^ A PSMA-Nb was conjugated to the cytotoxic drug doxorubicin, which demonstrated specific internalization into PSMA-expressing cells and induction of tumor growth inhibition following doxorubicin release.^22^ Moreover, anti-PSMA nanobodies have recently been applied to develop CAR-T cells, and their efficacy against prostate cancer cells has been confirmed in different studies.^23,24^

 Because of their single-gene format and the lack of post-translational modifications, nanobodies can be efficiently expressed in microbial systems. However, the expression of these proteins in more costly and demanding systems, such as mammalian cells, may be considered, particularly for complex nanobody fusion molecules. Regardless of the protein production goal, it is necessary to optimize parameters affecting three distinct steps: expression, solubilization, and purification. Several efforts have been made to optimize recombinant protein expression, such as changing the host strain and culture parameters such as IPTG concentration, incubation time, and temperature.^25^ IPTG, an artificial inducer of the lactose operon, has been used at 0.25-1.5 mM final concentrations.^26^ Since this chemical is potentially toxic for host cells, its concentration in the culture media should be adjusted in each experiment.^27^ Rosetta host strains are BL21 derivatives developed for improving the expression of eukaryotic proteins which contain codons rarely used in *E. coli*.^28^ The periplasmic expression of anti-TAG72 nanobodies in Rosetta-Gami2 yielded 0.3 to 0.6 mg/l of bacterial culture medium.^29^ Chao *et al. *found that OmpA and PelB signal peptides could enhance the solubility of an anti-GFP nanobody while preserving its bioactivity.^30^ To obtain higher yields of an active single-domain antibody, Bao *et al. *expressed a nanobody against human beta-2-microglobulin in the inclusion body form. By optimizing the dilution refolding conditions, they could purify 106.2 mg of correctly refolded nanobody from 1 L of *E. coli* culture.^31^ Likewise, Maggi *et al. *compared four different methods for the expression and extraction of camelid nanobodies as the insoluble inclusion bodies, and reported that classical inclusion bodies and its extraction based on a urea-mediated method produced yields of 60–70 mg/L.^32^ The final yield of PSMA-Nb was approximately 84 mg/L, which is about 30% higher than the yield reported by Maggi *et al. *Although inclusion body expression enables high yields, it requires carefully controlled denaturation and refolding to minimize risks such as aggregation, fragmentation, and loss of biological activity under harsh conditions. However, with optimized and scalable refolding protocols, inclusion body expression does not hinder scale-up or clinical translation.

 Pathological advancements have improved the accuracy and precision of prostate cancer diagnosis. Among these, IHC has established a niche for itself due to its critical function in detecting prostate cancer.^33^ Accurate IHC-based diagnosis may improve the prognosis and treatment of prostate cancer, resulting in better clinical results. The IHC staining can play a critical role in either confirming or ruling out the presence of cancer, which influences the subsequent therapeutic interventions.^34^ Moreover, the role of PSMA in personalizing prostate cancer therapy is still the focus of several clinical trials. For example, 24 out of the 61 PET studies for prostate cancer currently registered on ClinicalTrials.gov are PSMA-based.^35^ In order to help clinicians select the best imaging method as well as therapy for their patients, PSMA immunohistochemical evaluation should be studied further as a prognostic marker in males with metastatic PCa. PSMA-based strategies will play a critical role in the evolving diagnostic and therapeutic landscape of patients with PCa as our knowledge of PSMA’s role in prostate carcinogenesis and molecular techniques becomes more refined.^36^ Unlike prior anti-PSMA nanobody reports focused on *in vivo *imaging, we established a quantitative FFPE-IHC workflow on human tissue and demonstrated direct concordance with a commercial antibody under matched conditions; based on the available literature, this represents the first application of a PSMA-directed nanobody for standardized, quantitative IHC in FFPE sections, thereby extending nanobody utility from imaging to a pathology-ready diagnostic modality. Importantly, IHC and imaging are complementary modalities: IHC provides morphology-resolved, cell- and subcellular-level localization on tissue sections, while imaging offers whole-body data essential for staging and monitoring treatment response. These distinct yet synergistic applications together enable more comprehensive PSMA profiling. Novel nanobody-based technologies possess the potential to improve IHC, facilitating personalized medicine and enhancing our understanding of the disease at molecular and protein levels.^37^ However, regulatory requirements for nanobody-based clinical diagnostics including analytical validation, batch-to-batch consistency, and long-term stability must be rigorously addressed to enable their widespread clinical adoption. Future studies with larger, statistically appropriate sample sizes are needed to robustly validate the utility of PSMA-Nb in IHC. Although a conservative two-sample pooled-SD formulation was applied, the standardized effects remained “huge” (Hedges g ≈ 6.25 for PSMA-Nb and ≈ 4.65 for the commercial antibody), underscoring a robust tumor–normal contrast that will be further corroborated in a planned validation cohort using paired-difference estimates and 95% CIs.

 The strong and specific PSMA-Nb signal in tumor tissue is consistent with the well-documented upregulation of cell-surface PSMA in malignant prostatic epithelium, potentially influenced by androgen-responsive regulation,^38,39^ whereas minimal staining in matched normal tissue reflects low basal expression largely confined to luminal epithelial cells.^40^ These features provide a biological rationale for the high tumor-to-normal contrast observed in our quantitative FFPE-IHC assay. At the molecular level, the compact, single-domain architecture of the JVZ-007–derived nanobody facilitates epitope access in FFPE sections and efficient recognition despite formalin-induced crosslinking, consistent with binding to an extracellular/apical epitope. This interpretation aligns with our orthogonal readouts: ELISA dose–response; and flow cytometry showing robust PSMA-Nb binding in LNCaP (PSMA + ; 68.5% positive) and a substantially lower signal in DU145 (PSMA-; 2.35% positive) (Figure 6B); and quantitative FFPE-IHC on matched tumor–normal sections. A paired comparison indicated no inter-reagent difference. Together, these findings explain the robust tumor-to-normal separation and the staining intensity comparable to conventional antibodies.

 Key challenges in moving from IHC to *in vivo* applications of nanobodies include short half-life, immunogenicity and lack of effector functions. Rapid clearance remains a key constraint, as nanobodies are rapidly filtered renally.^41^ Although nanobodies are intrinsically low in immunogenicity, they can still elicit immune responses in therapeutic settings, necessitating humanization for clinical development. Despite their high target specificity and favorable properties, monomeric nanobodies inherently lack Fc-mediated effector functions such as antibody-dependent cellular cytotoxicity, limiting their direct utility in therapeutic contexts requiring immune cell recruitment or target cell lysis.^42^

## Conclusion

 In the present study, we sought to optimize the PSMA-Nb expression in quantities suitable for diagnostic applications. *E. coli* Rosetta (DE3) cells produced the highest yield of PSMA-Nb when induced with 1 mM IPTG at 37°C for 16 h, resulting in a protein yield that is cost-effective for large-scale production. Moreover, IHC analysis showed significant differences in the expression level of the PSMA antigen between normal and tumor-derived prostate biopsies. Due to its small molecular size, high production yield, and specific recognition of PSMA antigen in tumor tissues, this PSMA-Nb could be regarded as a diagnostic tool for prostate cancer.

## Competing Interests

 The authors declare that they have no competing interests.

## Data Availability of Statement

 All authors declare that the data generated or analyzed during this study are included in this published article and its additional file.

## Ethical Approval

 This study was approved by the. Ethics Committee of Pasteur Institute of Iran (IR.PII.REC.1399.069).

## Supplementary Files

Supplementary file 1 contains Table S1.

## References

[1] Sung H, Ferlay J, Siegel RL, Laversanne M, Soerjomataram I, Jemal A (2021). Global Cancer Statistics 2020: GLOBOCAN Estimates of Incidence and Mortality Worldwide for 36 Cancers in 185 Countries. CA Cancer J Clin.

[2] Barani M, Sabir F, Rahdar A, Arshad R, Kyzas GZ. Nanotreatment and Nanodiagnosis of Prostate Cancer: Recent Updates. Nanomaterials (Basel) 2020;10(9). doi: 10.3390/nano10091696. PMC7559844 32872181

[3] Bouchelouche K, Choyke PL, Capala J (2010). Prostate specific membrane antigen- a target for imaging and therapy with radionuclides. Discov Med.

[4] Sengupta S, Asha Krishnan M, Chattopadhyay S, Chelvam V (2019). Comparison of prostate-specific membrane antigen ligands in clinical translation research for diagnosis of prostate cancer. Cancer Rep (Hoboken).

[5] Debnath S, Zhou N, McLaughlin M, Rice S, Pillai AK, Hao G, et al. PSMA-Targeting Imaging and Theranostic Agents-Current Status and Future Perspective. Int J Mol Sci 2022;23(3). doi: 10.3390/ijms23031158. PMC8835702 35163083

[6] Jovčevska I, Muyldermans S (2020). The Therapeutic Potential of Nanobodies. BioDrugs.

[7] Siegel RL, Miller KD, Fuchs HE, Jemal A (2022). Cancer statistics, 2022. CA Cancer J Clin.

[8] Hu Y, Liu C, Muyldermans S (2017). Nanobody-Based Delivery Systems for Diagnosis and Targeted Tumor Therapy. Front Immunol.

[9] Kastelic D, Frković-Grazio S, Baty D, Truan G, Komel R, Pompon D (2009). A single-step procedure of recombinant library construction for the selection of efficiently produced llama VH binders directed against cancer markers. J Immunol Methods.

[10] Rosano GL, Ceccarelli EA (2014). Recombinant protein expression in Escherichia coli: advances and challenges. Front Microbiol.

[11] Chatalic KL, Veldhoven-Zweistra J, Bolkestein M, Hoeben S, Koning GA, Boerman OC (2015). A Novel ¹¹¹In-Labeled Anti-Prostate-Specific Membrane Antigen Nanobody for Targeted SPECT/CT Imaging of Prostate Cancer. J Nucl Med.

[12] Lu RM, Hwang YC, Liu IJ, Lee CC, Tsai HZ, Li HJ (2020). Development of therapeutic antibodies for the treatment of diseases. J Biomed Sci.

[13] Maali A, Gholizadeh M, Feghhi-Najafabadi S, Noei A, Seyed-Motahari SS, Mansoori S (2023). Nanobodies in cell-mediated immunotherapy: On the road to fight cancer. Front Immunol.

[14] Yang EY, Shah K (2020). Nanobodies: Next Generation of Cancer Diagnostics and Therapeutics. Front Oncol.

[15] Bakherad H, Farahmand M, Setayesh N, Ebrahim-Habibi A (2020). Engineering an anti-granulocyte colony stimulating factor receptor nanobody for improved affinity. Life Sci.

[16] Hosseindokht M, Bakherad H, Zare H (2021). Nanobodies: a tool to open new horizons in diagnosis and treatment of prostate cancer. Cancer Cell Int.

[17] Omidfar K, Shirvani Z (2012). Single domain antibodies: a new concept for epidermal growth factor receptor and EGFRvIII targeting. DNA Cell Biol.

[18] De Vos J, Devoogdt N, Lahoutte T, Muyldermans S (2013). Camelid single-domain antibody-fragment engineering for (pre)clinical in vivo molecular imaging applications: adjusting the bullet to its target. Expert Opin Biol Ther.

[19] Dumoulin M, Conrath K, Van Meirhaeghe A, Meersman F, Heremans K, Frenken LG (2002). Single-domain antibody fragments with high conformational stability. Protein Sci.

[20] Hrynchak I, Santos L, Falcão A, Gomes CM, Abrunhosa AJ. Nanobody-Based Theranostic Agents for HER2-Positive Breast Cancer: Radiolabeling Strategies. Int J Mol Sci 2021;22(19). doi: 10.3390/ijms221910745. PMC8509594 34639086

[21] Rosenfeld L, Sananes A, Zur Y, Cohen S, Dhara K, Gelkop S (2020). Nanobodies Targeting Prostate-Specific Membrane Antigen for the Imaging and Therapy of Prostate Cancer. J Med Chem.

[22] Heidenreich A, Bastian PJ, Bellmunt J, Bolla M, Joniau S, van der Kwast T (2014). EAU guidelines on prostate cancer Part II: Treatment of advanced, relapsing, and castration-resistant prostate cancer. Eur Urol.

[23] Hassani M, Hajari Taheri F, Sharifzadeh Z, Arashkia A, Hadjati J, van Weerden WM (2020). Engineered Jurkat Cells for Targeting Prostate-Specific Membrane Antigen on Prostate Cancer Cells by Nanobody-Based Chimeric Antigen Receptor. Iran Biomed J.

[24] Hassani M, Hajari Taheri F, Sharifzadeh Z, Arashkia A, Hadjati J, van Weerden WM (2019). Construction of a chimeric antigen receptor bearing a nanobody against prostate a specific membrane antigen in prostate cancer. J Cell Biochem.

[25] Chhetri G, Kalita P, Tripathi T (2015). An efficient protocol to enhance recombinant protein expression using ethanol in Escherichia coli. MethodsX.

[26] Shafiee F, Moazen F, Rabbani M, Mir Mohammad Sadeghi H (2015). Optimization of the Expression of Reteplase in Escherichia coli TOP10 Using Arabinose Promoter. Jundishapur J Nat Pharm Prod.

[27] Kosinski MJ, Rinas U, Bailey JE (1992). Isopropyl-β-d-thiogalactopyranoside influences the metabolism of Escherichia coli. Applied Microbiology and Biotechnology.

[28] Aguirre-López B, Cabrera N, de Gómez-Puyou MT, Perez-Montfort R, Gómez-Puyou A (2017). The importance of arginine codons AGA and AGG for the expression in E coli of triosephosphate isomerase from seven different species. Biotechnol Rep (Amst).

[29] Sharifzadeh Z, Rahbarizadeh F, Shokrgozar MA, Ahmadvand D, Mahboudi F, Rahimi Jamnani F (2013). Development of oligoclonal nanobodies for targeting the tumor-associated glycoprotein 72 antigen. Mol Biotechnol.

[30] Chao S, Liu Y, Ding N, Lin Y, Wang Q, Tan J (2022). Highly Expressed Soluble Recombinant Anti-GFP VHHs in Escherichia coli via Optimized Signal Peptides, Strains, and Inducers. Front Mol Biosci.

[31] Bao X, Xu L, Lu X, Jia L (2016). Optimization of dilution refolding conditions for a camelid single domain antibody against human beta-2-microglobulin. Protein Expr Purif.

[32] Maggi M, Scotti C (2017). Enhanced expression and purification of camelid single domain VHH antibodies from classical inclusion bodies. Protein Expr Purif.

[33] Kohale MG, Dhobale AV, Bankar NJ, Noman O, Hatgaonkar K, Mishra V (2023). Immunohistochemistry in pathology: A review. Journal of Cellular Biotechnology.

[34] Mandel P, Wenzel M, Hoeh B, Welte MN, Preisser F, Inam T (2021). Immunohistochemistry for Prostate Biopsy-Impact on Histological Prostate Cancer Diagnoses and Clinical Decision Making. Curr Oncol.

[35] von Eyben FE, Baumann GS, Baum RP (2018). PSMA diagnostics and treatments of prostate cancer become mature. Clin Transl Imaging.

[36] Cimadamore A, Cheng M, Santoni M, Lopez-Beltran A, Battelli N, Massari F (2018). New Prostate Cancer Targets for Diagnosis, Imaging, and Therapy: Focus on Prostate-Specific Membrane Antigen. Front Oncol.

[37] Fridy PC, Farrell RJ, Molloy KR, Keegan S, Wang J, Jacobs EY (2024). A new generation of nanobody research tools using improved mass spectrometry-based discovery methods. J Biol Chem.

[38] O’Keefe DS, Bacich DJ, Huang SS, Heston WDW (2018). A Perspective on the Evolving Story of PSMA Biology, PSMA-Based Imaging, and Endoradiotherapeutic Strategies. J Nucl Med.

[39] Wang L, Lu C, Wang X, Wu D (2025). PSMA targeted Therapy: From molecular mechanisms to clinical breakthroughs in castration-resistant prostate cancer. Eur J Med Chem.

[40] Silver DA, Pellicer I, Fair WR, Heston WD, Cordon-Cardo C (1997). Prostate-specific membrane antigen expression in normal and malignant human tissues. Clin Cancer Res.

[41] Hoefman S, Ottevaere I, Baumeister J, Sargentini-Maier ML (2015). Pre-Clinical Intravenous Serum Pharmacokinetics of Albumin Binding and Non-Half-Life Extended Nanobodies®. Antibodies [Internet].

[42] Rossotti MA, Bélanger K, Henry KA, Tanha J (2022). Immunogenicity and humanization of single-domain antibodies. Febs j.

