# Supplementary material for: Optimizing Expression of a Llama-Based Anti-PSMA Nanobody in Escherichia Coli and Its Application in Immunohistochemistry of Prostate Cancer Tissues
Source: Adv Pharm Bull. 2025 Dec 23;16(1):62–73. doi: 10.34172/apb.025.45447 (PMC13408249; doi:10.34172/apb.025.45447)
Supplement: Supplementary file 1 — contains Table S1. [file apb-16-62-s001.pdf]

Adv Pharm Bull 2026;16(1):62-S1
doi:10.34172/apb.025.45447
https://apb.tbzmed.ac.ir

Table S1. Summarizing the optimization expression, purification and IHC protocols

	Step	Key conditions/parameters
Expression of recombinant PSMA-Nb	Host & selection	*E. coli* Rosetta (DE3); Amp 100 µg/mL, *E. coli* Rosetta (Gami2); Kan 50 µg/mL
	Induction	OD600= 0.6–0.8 → IPTG 1 mM, 37 °C, 16 h, 180–220 rpm
	Harvest	5,000 × g, 10 min, 4 °C
	Lysis buffer	50 mM NaH_2PO_4, 300 mM NaCl, 10 mM imidazole→ resuspended lysis buffer in a cell pellet of bacterial cultures to check the presence of PSMA-Nb protein (soluble fraction) and pellet fractions (inclusion bodies)
	Sonication	30% -70% amplitude (6× 10 s On / 20 s Off at 200-300 W), on ice (≤10 °C)
	Clarification	15,000 × g, 25 min, 4 °C
Purification of recombinant PSMA-Nb	Ni-NTA	Wash 10–20 mM imidazole; Urea: 8→6→4→2→0 M<br>Elution: 250–500 mM imidazole
	Buffer-exchange	PBS pH 7.4 (10–20 CV), 10–30 kDa MWCO concentrator
	QC	SDS-PAGE/Western; ELISA binding; flowcytometry (LNCaP$^+$ vs DU145$^-$)
	Storage	4 °C ≤1 week; −80 °C long-term (aliquoted)
PSMA Immunohistochemistry (FFPE)	Sections	FFPE, 5 µm on Superfrost Plus slides
	Deparaffinization & rehydration	Dewax → graded ethanols → water (standard sequence)
	Antigen retrieval	TBS buffer, microwave 800 W, 20 min; pH 7.6, cool to RT
	Blocking	Serum-free protein block (Agilent), 30 min, RT
	Primary reagents	PSMA-Nb at 1 or 3 µg/section; PSMA1 (Biorbyt) 1:100 as positive control; incubate 24 h at 2–8 °C
	Washes	PBS, 3 ×
	Secondary detection	For PSMA-Nb: MonoRab™ anti-camelid VHH, FITC-conjugated (GenScript) and; and for PSMA1: Goat anti-rabbit IgG (H+L), FITC-conjugated (Biorbyt); 1 h, RT, dark

	Counterstain & mounting	DAPI, wash PBS, mount in glycerol:PBS
	Imaging	Olympus microscope; capture FITC/DAPI images under identical settings across slides
	Quantification	Compute fractional fluorescent area per mm^2 (total FITC-positive area ÷ total section area) as average protein level (mm^{-2})
